# Structures of the SARS-CoV-2 spike glycoprotein and applications for novel drug development

**DOI:** 10.3389/fphar.2022.955648

**Published:** 2022-08-09

**Authors:** Xiao-Huan Liu, Ting Cheng, Bao-Yu Liu, Jia Chi, Ting Shu, Tao Wang

**Affiliations:** School of Biological Science, Jining Medical University, Jining, China

**Keywords:** COVID-19, spike glycoprotein, small-molecule inhibitors, drug development, computer-aided drug development

## Abstract

COVID-19 caused by SARS-CoV-2 has raised a health crisis worldwide. The high morbidity and mortality associated with COVID-19 and the lack of effective drugs or vaccines for SARS-CoV-2 emphasize the urgent need for standard treatment and prophylaxis of COVID-19. The receptor-binding domain (RBD) of the glycosylated spike protein (S protein) is capable of binding to human angiotensin-converting enzyme 2 (hACE2) and initiating membrane fusion and virus entry. Hence, it is rational to inhibit the RBD activity of the S protein by blocking the RBD interaction with hACE2, which makes the glycosylated S protein a potential target for designing and developing antiviral agents. In this study, the molecular features of the S protein of SARS-CoV-2 are highlighted, such as the structures, functions, and interactions of the S protein and ACE2. Additionally, computational tools developed for the treatment of COVID-19 are provided, for example, algorithms, databases, and relevant programs. Finally, recent advances in the novel development of antivirals against the S protein are summarized, including screening of natural products, drug repurposing and rational design. This study is expected to provide novel insights for the efficient discovery of promising drug candidates against the S protein and contribute to the development of broad-spectrum anti-coronavirus drugs to fight against SARS-CoV-2.

## 1 Introduction

The 2019 novel coronavirus disease (COVID-19) caused by severe acute respiratory syndrome coronavirus 2 (SARS-CoV-2) has rapidly spread to more than 210 countries and has become a serious threat to global public health (Jiang et al., 2020; Lai et al., 2020). To date, the globe is still struggling with COVID-19. Coronaviruses (CoVs) can infect humans and animals and cause a variety of diseases, such as fever, severe respiratory illness and pneumonia, threatening human health and public safety. CoVs are mainly divided into four genera, α-CoV, beta-CoV, gamma-CoV, and delta-CoV (Ou et al., 2020). During the past 2decades, β-CoVs have caused three severe zoonotic outbreaks: severe acute respiratory syndrome-CoV (SARS-CoV) in 2003, Middle East respiratory syndrome-CoV (MERS-CoV) in 2012, and newly emerged SARS-CoV-2 in late 2019. To date, several promising antiviral medicines, such as remdesivir (Schooley et al., 2021), molnupiravir (Holman et al., 2021), and paxlovid (ritonavir/PF-07321332) (Zhao et al., 2021), have been developed or approved for marketing; unfortunately, no specific medicine or standard treatment has been developed yet to combat COVID-19.

Although the physiology-based approach is a traditional and proven drug discovery paradigm for the development of novel drugs, emerging computer-aided drug development has become a promising alternative to accelerate the modern discovery process (Yadav et al., 2020; Gentile et al., 2021). These *in silico* strategies could very effectively identify novel active scaffolds for a validated target. Therefore, target identification (i.e., one or more targets) has become a key starting point for a successful drug discovery project, which is also true for the development of pancoronavirus (HCoV) antiviral drugs (Liu et al., 2021).

**FIGURE 1:**
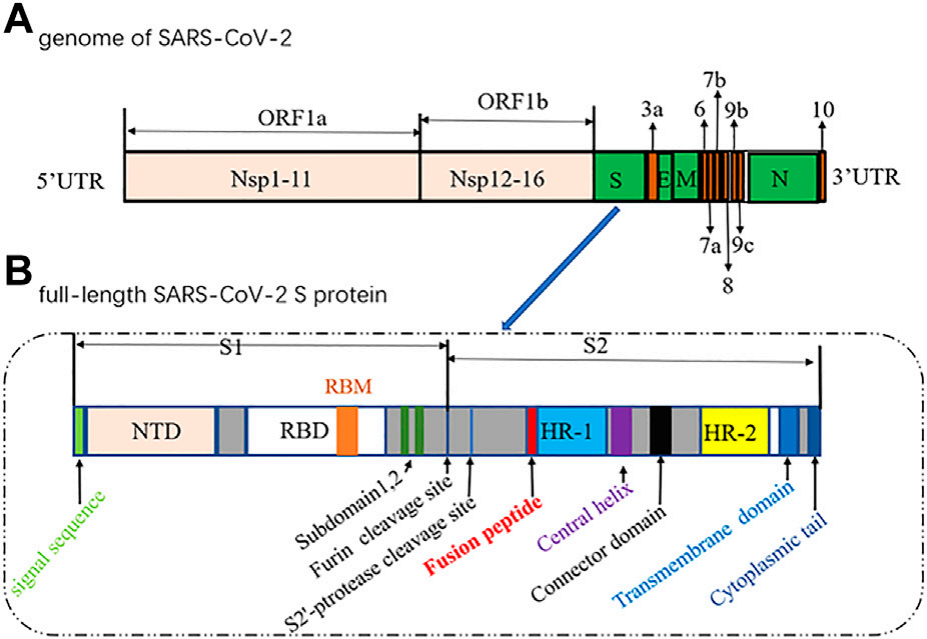
Diagrams of the genome of SARS-CoV-2 (1) and the full-length SARS-CoV-2 S protein (2).(NTD, N-terminal domain; RBM, receptor-binding motif; RBD, receptor-binding domain; HR, heptad repeat).

The genome of SARS-CoV-2 contains two large overlapping open reading frames (Figure 1A, ORF1a and ORF1b) encoding 16 non-structural proteins (Nsp1 to 16), along with open reading frames encoding four structural proteins (spike (S), membrane (M), envelope (E), and nucleocapsid (N)) and nine accessory proteins (Chan et al., 2020; Pillay, 2020). The trimeric S protein (∼180 kDa, Figure 1B), consisting of the S1 and S2 subunit, is crucial for the virus to enter the cell. In particular, S1 contains a receptor-binding domain (RBD) that binds to angiotensin-converting enzyme 2 (ACE2) to initiate the entry of the virus into cells (Wang et al., 2020). Considering that the SARS-CoV-2 viral life cycle starts with the binding of the S-RBD to the host ACE2 receptor, the S protein, especially the S-RBD, is considered a key molecular target for the development of vaccines, therapeutic agents, and diagnostic methods against COVID-19 (Pandey et al., 2021; Souza et al., 2021; Tan et al., 2021; Zahradník et al., 2021; Gyebi et al., 2022).

Given the importance of the S protein in the context of the COVID-19 pandemic, in this review, the molecular features of the S protein of SARS-CoV-2 are highlighted. Additionally, computational tools available for the treatment of COVID-19 were also provided. Finally, recent advances in the novel development of antivirals against the S protein are summarized. Taken together, this study provides an essential foundation for the design and development of efficient antiviral agents based on the SARS-CoV-2 S protein.

## 2 Structures and functions of the SARS-CoV-2 spike glycoprotein

### 2.1 S protein: A key target for antivirals

The surface transmembrane spike glycoprotein S is a typical class I viral fusion protein that is responsible for viral attachment to host cells, subsequent virus–cell membrane fusion and humoral and cell-mediated response induction (Hatmal et al., 2020). The overall structure of the SARS-CoV-2 S protein is quite similar to that of SARS-CoV S; the main conformational difference lies in the position of the receptor-binding domain (RBD) (Wrapp et al., 2020). ACE2 can bind more tightly to the SARS-CoV-2 S protein (with ∼15 nM affinity) than to the SARS-CoV S protein. This may help explain the enhanced pathogenicity of COVID-19 compared with that of SARS-CoV. The amino acid sequence identity of the S proteins of SARS-CoV-2 and SARS-CoV is approximately 77%, also indicating that they are closely related phylogenetically (Zhou et al., 2020).

**FIGURE 2:**
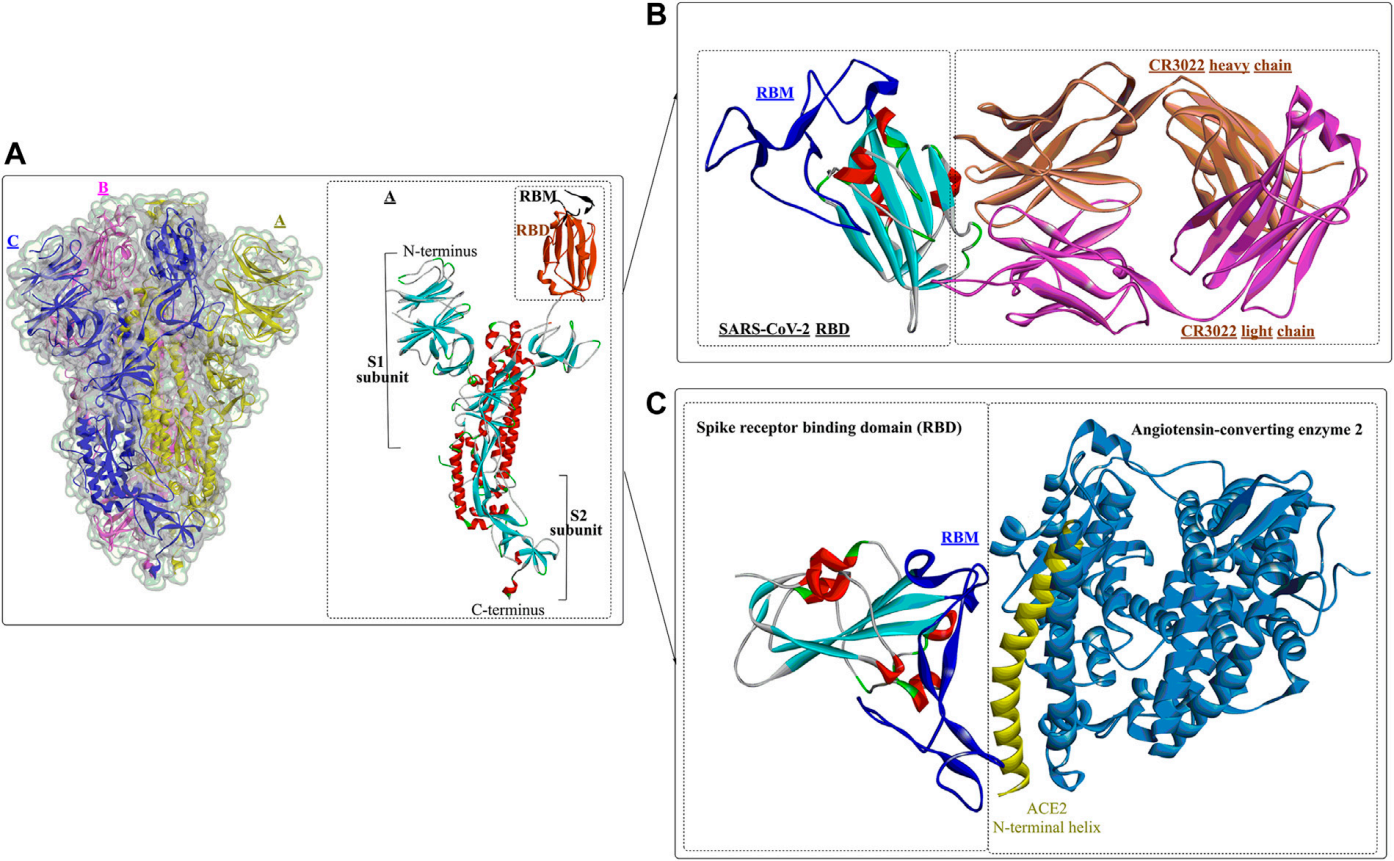
Crystal structure of prefusion SARS-CoV-2 spike glycoprotein (1, PDB ID: 6VSB), antibody CR3022 binding to the SARS-CoV-2 RBD (2, PDB ID: 6W41), and the SARS-CoV-2 spike receptor-binding domain bound with ACE2 (3, PDB ID: 6M0J).

Usually, when the S protein is processed and hydrolysed by one or multiple host proteases [e.g., furin and transmembrane protease serine protease-2 (TMPRSS-2)], it will lead to the formation of active and fusion-competent S protein (Ou et al., 2020). For binding to the host cell receptor, the RBD undergoes a transient hinge-like conformational change from the “down” conformation (receptor inaccessible) to the “up” conformation (receptor accessible) (Peng et al., 2020; Meirson et al., 2021). In coronaviruses, the fusion-competent S protein (Figure 2A)) usually forms a trimer carrying the receptor-binding subunit S1 (700 amino acids) and the membrane-fusion subunit S2 (600 amino acids). It is noted that an insertion of four amino acid residues at the junction of S1 and S2 of SARS-CoV-2 will generate a polybasic cleavage site (RRAR), which would greatly facilitate effective cleavage (Andersen et al., 2020; Coutard et al., 2020). For SARS-CoV and SARS-CoV-2 (Lan et al., 2020), angiotensin-converting enzyme 2 (ACE2) is required for binding to target cells (Figure 2C), while dipeptidyl peptidase 4 (DPP4) is the necessary cellular receptor of MERS-CoV (Raj et al., 2013).

The RBDs of SARS-CoV-2, MERS-CoV, and SARS-CoV are located in the S1 subunit (Du et al., 2017) and are composed of a core subdomain and a receptor-binding motif (RBM) mediating viral attachment to host cells. Differences in the RBM domains would lead to the use of different receptors in varying hosts (Lu et al., 2013; Wang et al., 2013). Upon binding to the receptor, the S1 subunit dissociates from the trimeric S protein and is then exposed to the other subunit, S2 (Benton et al., 2020)^,^ (Cai et al., 2020). The S2 subunit contains several important structural elements (Walls et al., 2016), including an N-terminal fusion peptide (FP), heptad repeat 1 (HR1), the central helix (CH), the connector domain (CD), heptad repeat 2 (HR2), the transmembrane region (TM), and the cytoplasmic tail (CT). FP can bind to the target cell membrane and, once bound, will induce S2 into a prehairpin state to connect the viral and cellular membranes. Then, 3 HR1 regions self-assemble into a trimeric coiled coil, and 3 HR2 regions fold into the interface of the HR1 inner core, forming a six-helix bundle (6-HB) structure (Yuan et al., 2017).

Wang et al. determined the crystal structure of the RBD of SARS-CoV-2 bound to the cell receptor ACE2 (Figure 2C), and the results revealed that the interaction modes resemble those of the SARS-CoV RBD (Lan et al., 2020). The binding site is composed of five-stranded antiparallel *β* sheets, several short connecting helices, and loops. Among these secondary structures, four pairs of disulfide bonds formed by eight cysteine residues were also identified, which are used for stabilizing the *β* sheets. Analysis of the critical residues associated with RBD binding revealed that a total of 16 residues in the RBD (Figure 2C in dark blue) might interact with the N-terminal helix of ACE2. Among these critical residues, hydrophilic interactions (13 hydrogen bonds and 3 salt bridges) are formed during binding to the ACE2 receptor. It is worth noting that in this study, no interactions between the N-acetyl-β-glucosaminide (NAG) glycans and SARS-CoV-2 RBD were found, although the glycan-RBD interaction is believed to be associated with the binding of the SARS-CoV RBD to ACE2 (Li et al., 2005).

In a recent study (Yuan et al., 2020), a highly conserved cryptic epitope in the RBD of SARS-CoV-2 and SARS-CoV was discovered, which could be recognized by the neutralizing antibody CR3022 (Figure 2B). The presence of a glycan would induce CR3022 to bind more tightly to SARS-CoV than SARS-CoV-2. Although this special domain is distal from the traditional RBD, it makes cross-reactive binding between SARS-CoV-2 and SARS-CoV possible. In particular, it was found that the binding epitope could be accessed by CR3022 only when at least two of the three RBDs on the trimeric S protein were in the “up” conformation.

### 2.2 Interactions between the S protein and ACE2

**FIGURE 3:**
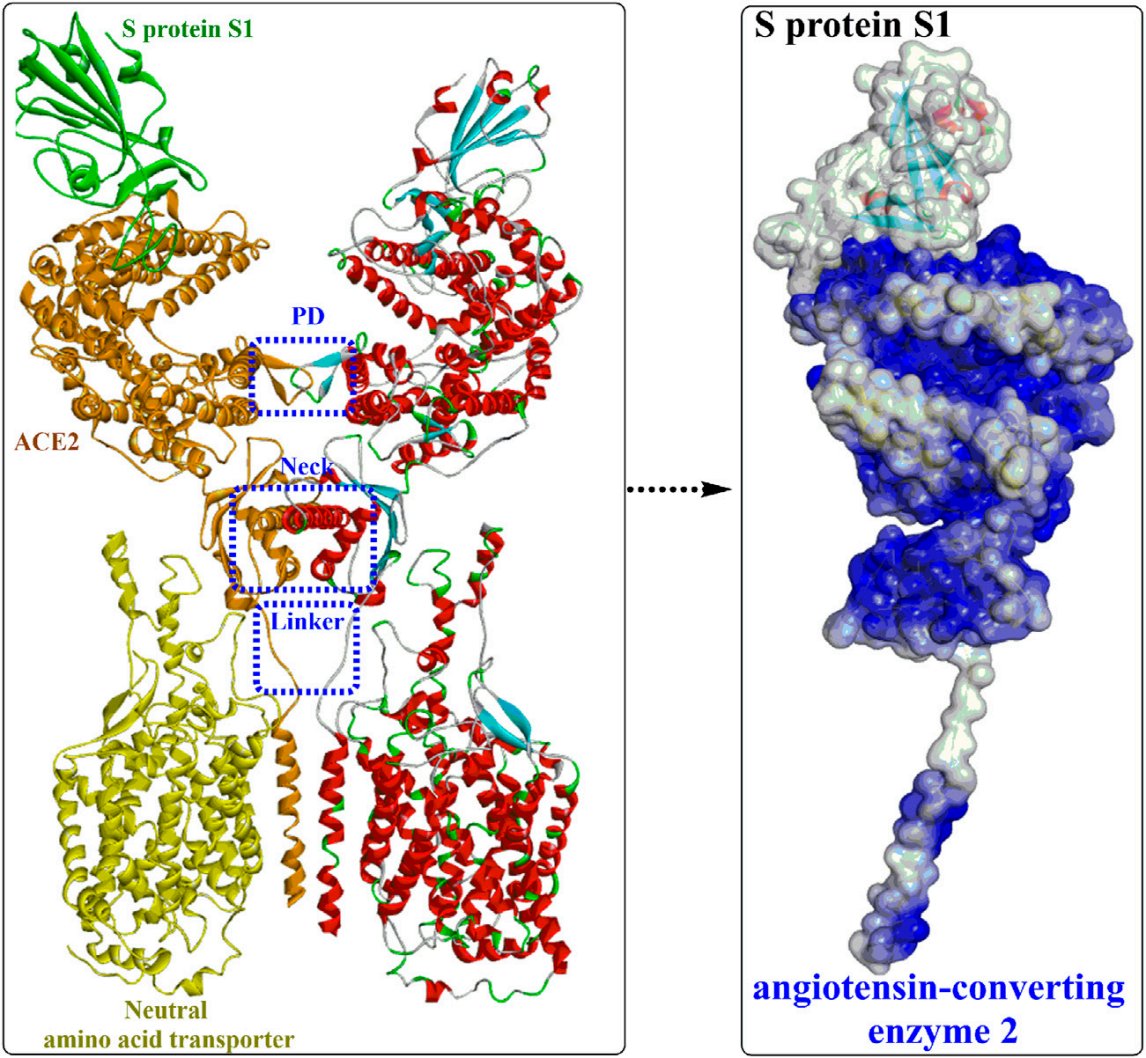
Overall structure of the RBD-ACE2-neutral amino acid transporter complex (PDB ID: 6M17).

Yan et al. (Yan et al., 2020) released the cryo-electron microscopy structures of full-length human ACE2 in the presence of the neutral amino acid transporter B0AT1 and the RBD of the S protein of SARS-CoV-2 (Figure 3). The ACE2-B0AT1 complex is assembled as a dimer of heterodimers (Figure 3A) with two critical functional domains, including an N-terminal peptidase domain (PD, residues 19–615) and a C-terminal collectrin-like domain (CLD) of ACE2. The RBD is recognized and directly binds to the PD of ACE2 mainly through polar interactions, and the homodimerization process is mediated by CLD (Song et al., 2018).

**FIGURE 4:**
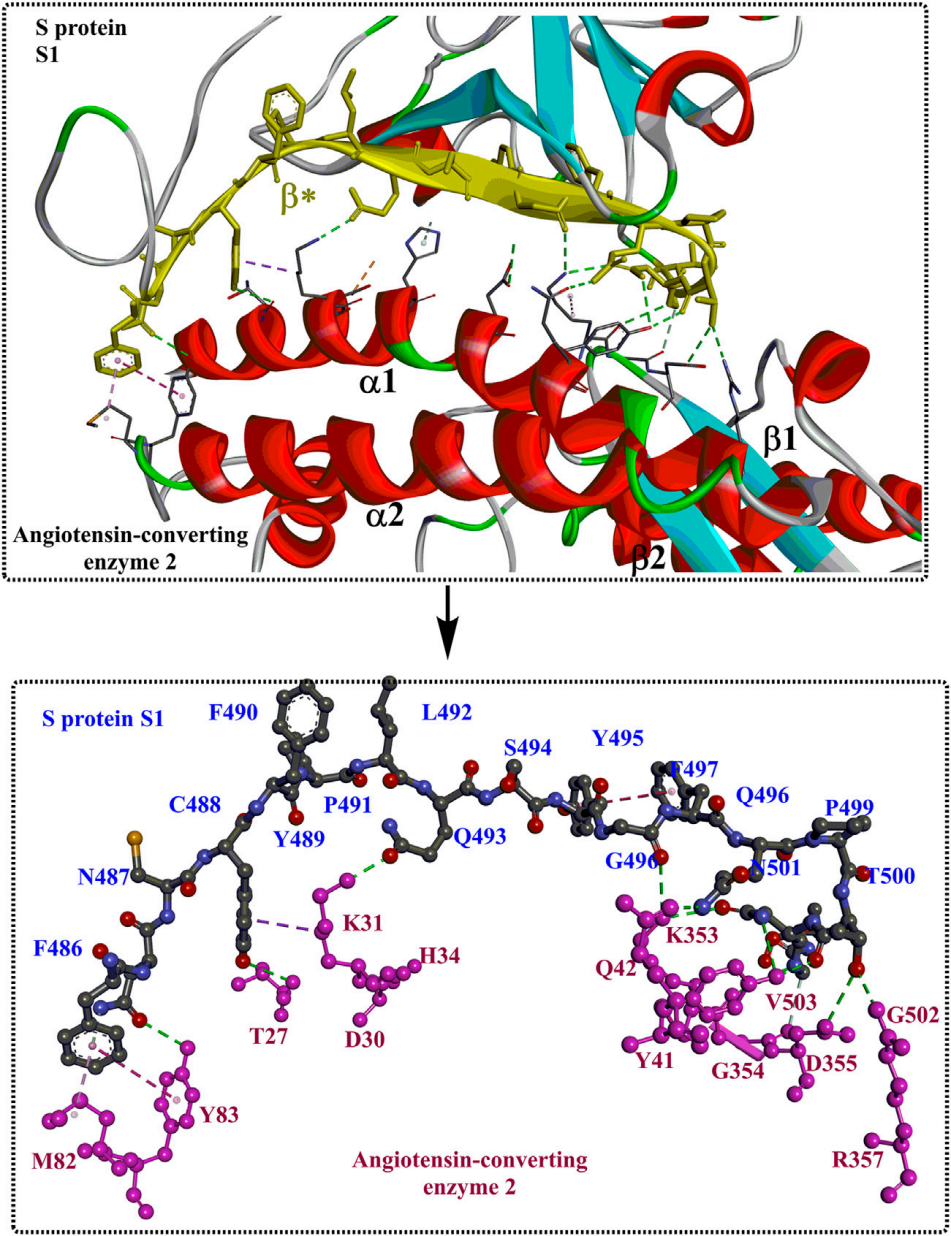
The interaction modes of S1 and ACE2 (PDB ID: 6M17).

Interactions between S1-RBD and ACE2 mainly occur in the region constructed by residues F486 to V503 (β*, coloured yellow). The two ends of the β* region strongly interact with the N- and C-termini of the α1 helix and certain areas on the α2 helix and *β*1 sheet. Moreover, the interaction can be further stabilized by interactions through several polar residues in the middle of α1 (Figure 4). At the N-terminus of α1, P499, T500, and N501 of the RBD form a network of H-bonds with Y41, Q42, K353, V503, G354, D355, and R357 from ACE2 (Figure 4B). In the middle of β*, Lys417 and Tyr453 of the RBD interact with Asp30 and His34 of ACE2, respectively (shown in green, Figure 4B). At the C-terminus of α1, Q493, C488 of the RBD is H-bonded to K31 and T27 of ACE2, respectively (shown in green, Figure 4B), whereas F486 of the RBD interacts with M82 and Y83 of ACE2 through van der Waals forces (shown in purple, Figure 4B). It is clear that those identified residues associated with the interactions with ACE2 would certainly make potential targets for inhibitors against virus replication. In addition, it was found that the T470-F490 loop (activated in the open state) and Q498-Y505 residues within the RBD domain of SARS-CoV-2 S act as viral determinants for the specific recognition of SARS-CoV-2 RBD by ACE2 (Xu et al., 2021).

### 2.3 Mutations in the spike protein and related interactions

Owing to the crucial role of the S protein in entering the cell for the virus, key mutations of the S protein might alter the virus’ infectivity, virulence, and antigenicity, leading to the reduced effectiveness of therapeutic antibodies and vaccines (Wang et al., 2021a; Wang et al., 2021b; Starr et al., 2021). Results showed that mutants (N501Y, E484K, and K417 N/T) with high mutation frequencies might have become the main genotypes for the spread of SARS-CoV-2 (Yi et al., 2021). Recent quantitative analysis of the stability of the ACE2-RBD complex for the Omicron variant (SARS-CoV-2 B.1.1.529) showed that its RBD could bind more strongly to the target human ACE2 protein than the original strain through increased hydrogen bonding interactions and a more buried solvent-accessible surface region (Lupala et al., 2022). This might help explain why 85% of previously characterized neutralization antibodies lost their efficacy against the new variant Omicron (Cao et al., 2022). Omicron (B.1.1.529) exhibits more than thirty amino acid mutations in the receptor-binding motif of the spike protein, and the increases in transmissibility and immune evasion have caused a challenging and threatening situation worldwide (Kannan et al., 2021; Meo et al., 2021).

**FIGURE 5:**
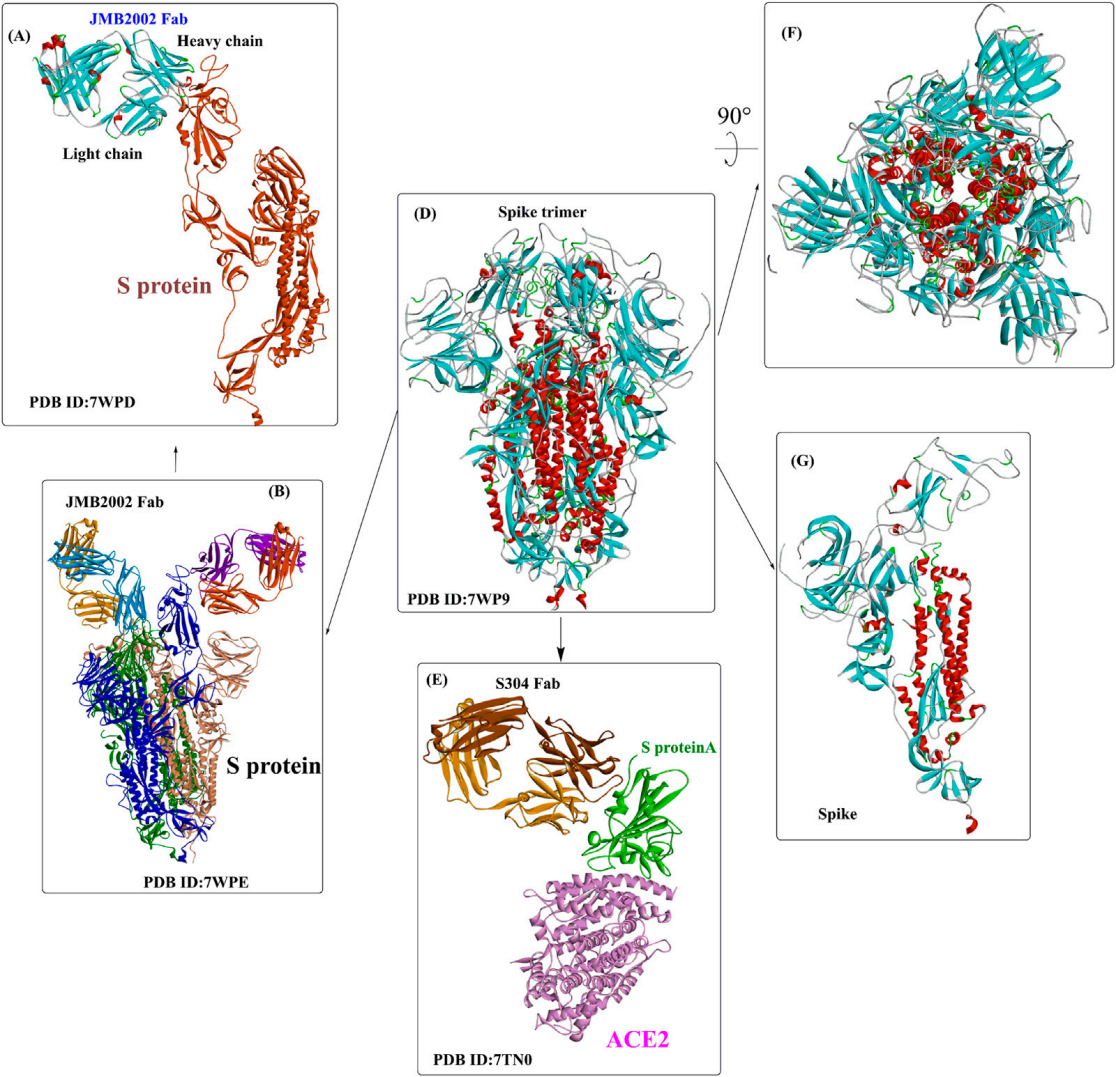
The interaction modes of the Omicron spike protein and ACE2.

**FIGURE 6:**
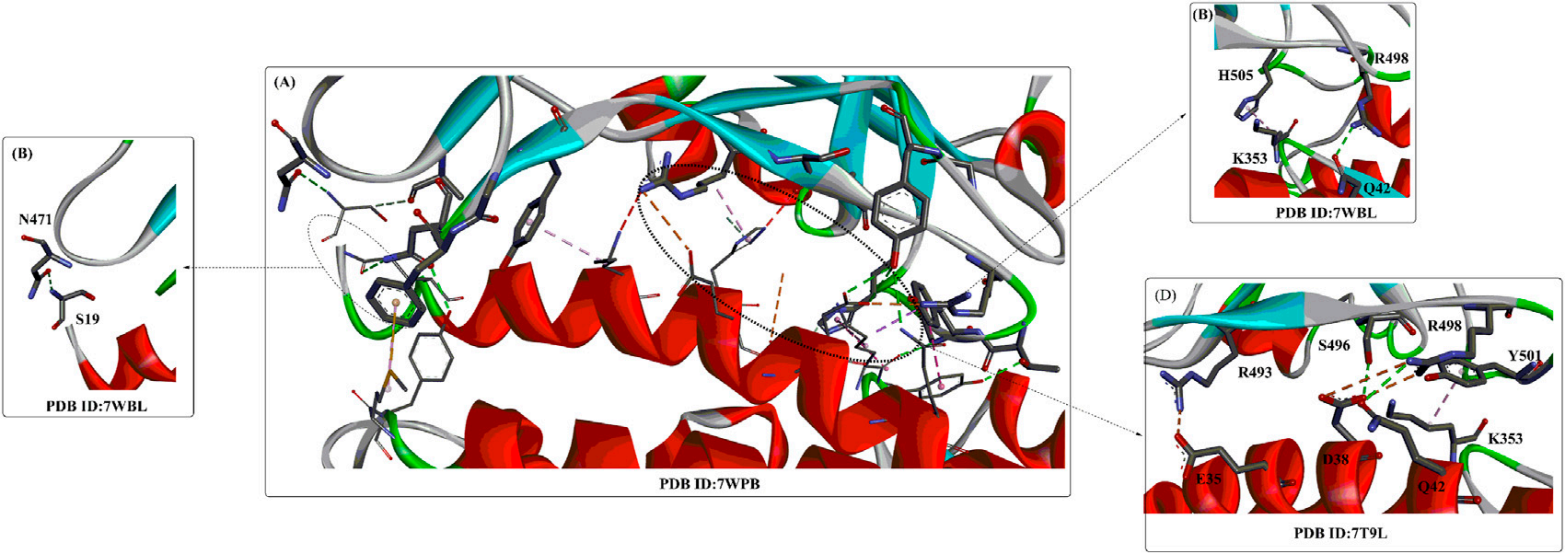
The interaction modes of the Omicron spike protein and ACE2.

Recently, several crystal structures of the Omicron spike trimer in complex with angiotensin-converting enzyme 2 (ACE2) or the therapeutic antibody (JMB 2002) (Han et al., 2022; Hong et al., 2022; McCallum et al., 2022; Yin et al., 2022) have been released. With 15 mutated residues, the overall structure of the Omicron ACE2-RBD complex is similar to that of the wild-type ACE2-RBD complex (Figure 5). Most Omicron mutations are located on the surface of the spike protein and change binding epitopes to many current antibodies. In the ACE2-binding site (Yin et al., 2022), compensating mutations strengthen RBD binding to ACE2, forming additional interactions with ACE2, including interactions from the RBD mutations N477 (hydrogen bonds), R493, Q496, R498 (hydrogen bonds), and Y501 (packing interactions) to ACE2 (Figure 6A). Moreover, RBD-RBD interactions from one of the two down RBDs to the up RBD were found, which might be capable of stabilizing the up conformation of the RBD. In contrast, the mutant residues (Omicron residues L371, P373, and F375) located at the entrance to the fatty acid–binding pocket could probably distort the pocket and destabilize the RBDs in the all closed-down conformation. All the mentioned interactions could further contribute to the higher affinity of Omicron. Similar findings also revealed new salt bridges, hydrogen bonds and π-stacking interactions formed by mutated residues R493, S496, R498 and Y501 in the RBD with ACE2 (Figure 6D) (Mannar et al., 2022). It was also found that the mutant residues N471 (H bond), H505 (van der Waals interactions), and R498 (salt bridge) also play important roles in hACE2 binding (Mannar et al., 2022).

### 2.4 N-terminal domain of the S protein of SARS-CoV-2

**FIGURE 7:**
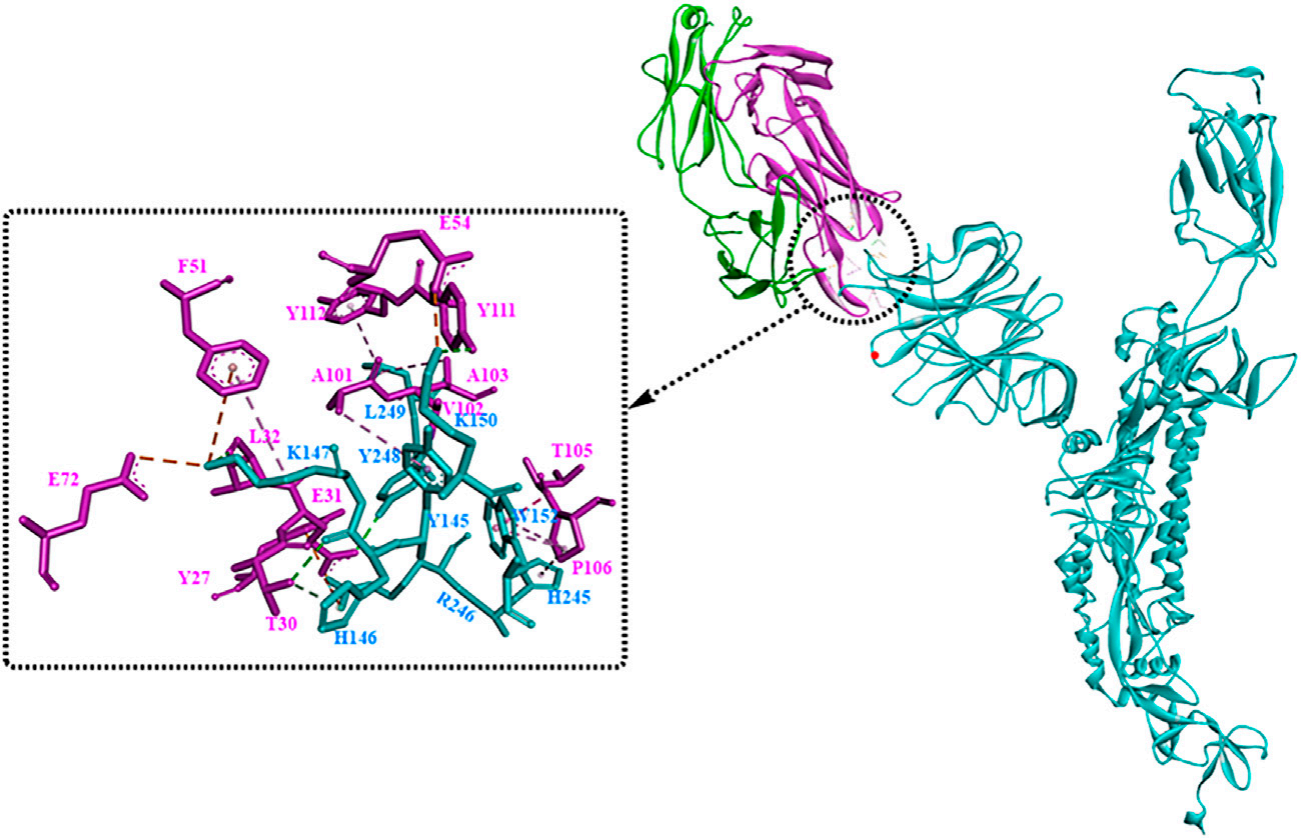
The interaction modes of the 4A8 and S-NTD complex (PDB:7C2L).

**FIGURE 8:**
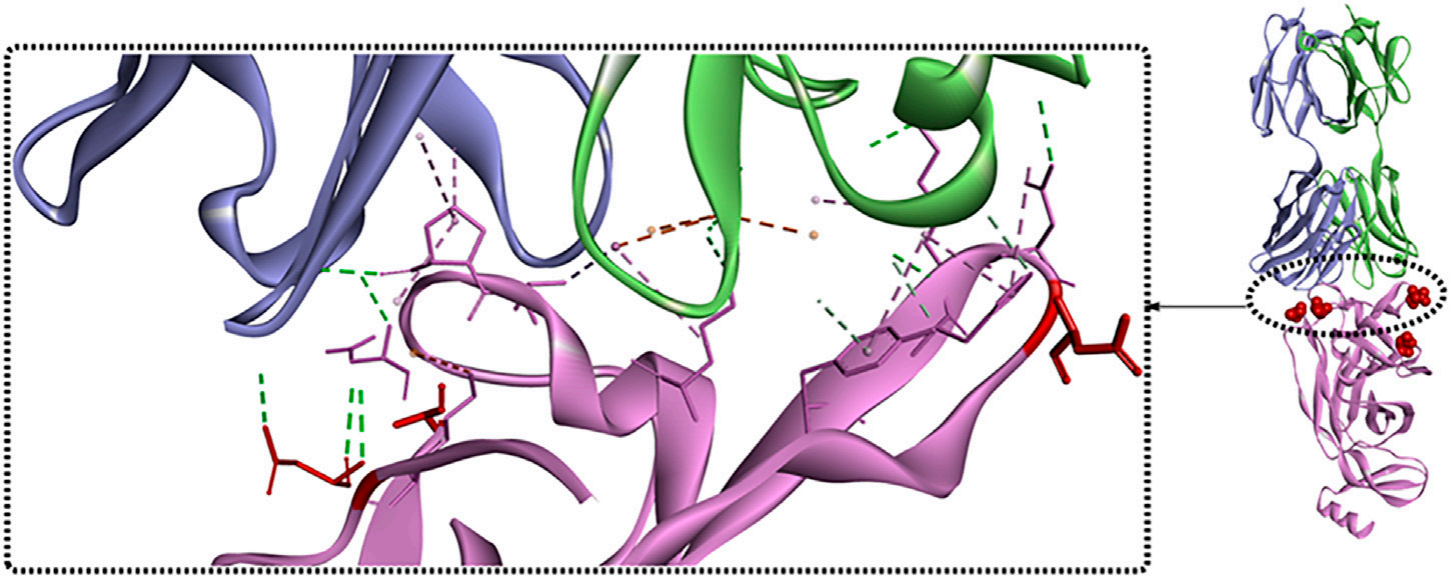
Crystal structure analysis of S-NTD bound to S2M28 Fab (PDB:7LY3).

It has been revealed that S1 consisted of the NTD and the RBD plays a critical role in the lifecycle of SARS-CoV-2. Though the detailed functions of NTD have not been well investigated, several studies showed that drug development against the NTD, especially the NTD-directed antibodies, might be another promising strategy (Ciuffreda et al., 2021; Di Gaetano et al., 2021; Schuurs et al., 2021). In a study by Chi et al. (2020), a neutralizing human antibody binding to the NTD of the S protein was developed and investigated. The biological results showed that the neutralizing capacity of 4A8 with EC_50_ of 0.61 mg/ml, moreover it could protect the ACE2-293T cells with an EC_50_ of 49 mg/ml. From the structure of the complex between 4A8 and S-NTD, it shows that the heavy chain of 4A8 mainly participates in binding to the NTD, on the contrary the light chain is away from the RBD. On this basis, it was estimated that 4A8 might play important functions in restraining the conformational changes of the S protein. Also, at the surface area of the 4A8-NTD interface critical residues including K147, Y248, K150, H146, R246, H245, W152, L129, N149, and so on could interact with 4A8 by means of H-bounds, salt bridges and hydrophilic interactions (Figure 7). In another study (Cerutti et al., 2021), structural analysis of seven potent NTD-directed neutralizing antibodies revealed a common highly electropositive binding site, which is formed by a mobile β-hairpin and several flexible loops including the critical residues glycans N17, N74, N122, and N149. Similarly, Matthew et al. also identified a supersite (site I, Figure 8), which could be recognized by all known NTD-specific neutralizing antibodies (McCallum et al., 2021). These studies indicate that potent NTD-directed neutralizing antibodies might probably target the single supersite.

## 3 Targets for novel drug development

Given that viral entry is mainly mediated by the trimeric spike protein, the S protein is considered a major therapeutic target for the treatment of SARS-CoV-2 infections. To interfere with the S protein-hACE2 interaction, neutralizing antibodies are usually the most traditional and functional strategies. However, inhibitors of the protein–protein interaction (PPI) between the S protein and hACE2 have recently drawn increasing attention for the development of potential antiviral agents to prevent viral attachment and cellular entry to control the ongoing COVID-19 pandemic (Tai et al., 2020; Bojadzic et al., 2021). In addition, although small molecular weight inhibitors (SMIs) are usually not considered potential candidates for PPI modulation, an increasing number of studies have revealed that SMIs could also be effective against certain PPIs (Scott et al., 2016; Risner et al., 2020; Pan et al., 2021).

Considering that anti-SARS-CoV-2 neutralizing antibodies have been extensively investigated (Ju et al., 2020; Cho et al., 2021; Wang et al., 2021c; Lucas et al., 2021), in this section, the recent drug development of novel small-molecule inhibitors (nonpeptide small molecules) that can interfere with viral entry or viral propagation is highlighted, including the discovery of natural products, drug repurposing, and novel drug development.

### 3.1 Computational tools developed for COVID-19 treatment

In addition to the traditional computational tools frequently used for rational drug development, such as Discovery Studio, Gold and AutoDock, several types of computational resources, tools and databases have recently been developed to investigate the ever-growing available data against COVID-19 and its related diseases.

(1) The D3Targets-SARS-CoV-2 web server (https://www.d3pharma.com/D3Targets-SARS-CoV-2/index.php.) is a webserver capable of predicting potential drug targets and identifying lead compounds against specific or multiple targets via structure-based virtual screening against COVID-19 (Shi et al., 2020). It provides two strategies for target prediction and virtual screening: the structure-based method (D3Pockets) and the ligand-based method (D3Similarity). The potential ligand-binding pockets is predicted by D3Pockets, and the docking process is performed with AutoDock Vina. By the end of 27-05-2021, 56 potential proteins (constructed by homology modelling or *de novo* prediction) involved in the whole process of virus life have been included.
(2) D3Similarity is a ligand-based method developed based on the molecular similarity evaluation between the submitted molecule(s) and the active compounds in the database (604 molecules) (Yang et al., 2021a). The 2D molecular similarity is evaluated by using Open Babel based on the Tanimoto coefficient (Tc) values between the SMILES of the input structure and the molecules in the database. The 3D molecular similarity was evaluated by using MolShaCS (Molecular Shape and Charge Similarity). D3Pockets is a web server developed for systematically exploring protein pocket dynamics based on either molecular dynamic simulation trajectories or conformational ensembles with large-scale conformational changes (Chen et al., 2019). Based on D3Pockets, the stability, continuity, and correlation of protein pockets could be investigated, and the results could also be visualized with PyMOL.
(3) CovidExpress (https://stjudecab.github.io/covidexpress) is an open-access database and interactive visualization tool for intuitive investigation of SARS-CoV-2-related transcriptomes, and we collected approximately 1,500 human bulk RNA-seq datasets from publicly available resources (Djekidel et al., 2021). It can be used to examine the relative gene expression levels in different tissues, cell lines, and especially the response to SARS-CoV-2. Based on this database, a series of commonly regulated genes (∼345 genes, 280 upregulated and 65 downregulated) in SARS-CoV-2-infected lung and nasal cells were identified, such as the interferon response genes *OASL TNF*, *IL1A*, and *CXCL10.*
(4) The COVID-19 Docking Server (http://ncov.schanglab.org.cn) is a web server that can be used for the prediction of the binding modes between COVID-19 targets and ligands (Kong et al., 2020). It provides a free and interactive tool for the prediction of COVID-19 target-ligand interactions and subsequent drug development. A total of 27 targets (e.g., spike protein, nucleocapsid protein, main protease, papain-like protease, and RNA-dependent RNA polymerase) involved in the virus life cycle were collected or constructed based on homology modelling and prepared for docking on the website. For different ligands, the implementation methods are different. For small molecular weight ligands, Open Babel is applied for format transformation and 3D coordinate generation, and AutoDock Vina is used for molecular docking. However, for macromolecular drugs (e.g., peptides and antibodies), CoDockPP is used as a docking engine with a multistage fast Fourier transform (FFT)-based strategy for both global docking and site-specific docking.
(5) MolAICal (https://molaical.github.io) was developed for the rational design of potential 3D drug structures in the 3D pockets of specific targets achieved by using a deep learning model and classical algorithms (Bai et al., 2021). It contains two main modules: one can employ the genetic algorithm, deep learning and the Vinardo score for rational drug design. The second module can use a deep learning generative model and molecular docking (achieved by AutoDock Vina) for virtual screening. The models used in this tool have been fully trained by different databases and methods. Several user-defined rules (e.g., Lipinski’s rule of five, synthetic accessibility) are also introduced for filtering out undesired hits.
(6) COVID19 db (http://hpcc.siat.ac.cn/covid19db or http://www.biomedicalweb.com/covid19db) is a user-friendly and open-access platform that integrates 95 COVID-19-related human transcriptomic datasets of 4,127 human samples across 13 body sites associated with exposure to 33 microbes and 33 drugs/agents in GEO and 39, 930 drug–target–pathway interactions among 2,037 drugs, 1,116 targets, and 207 pathways in DrugCentral and KEGG (Zhang et al., 2022). In addition, 14 different analytical applications (included in the differential expression and coexpression modules) and a web service tool are designed and integrated to analyse the integrated data or the obtained human transcriptomic data. Moreover, a drug discovery tool is provided for the identification of potential drugs and targets of COVID-19 and its related diseases at the whole transcriptomic level.

In addition to those mentioned above, some other computational tools have been developed to meet the urgent demand of the COVID-19 outbreak, which are listed in Table 1.

**TABLE 1:** Other computational tools developed for the analysis of COVID-19-related data.

Name	Functions	Website	Ref
Virus-CKB	(1) Target prediction; (2) platform of viral-associated computing resources; (3) drug development	https://www.cbligand.org/g/virus-ckb	Feng et al. (2021)
DINC-COVID	Ensemble docking with flexible SARS-CoV-2 proteins	http://dinc-covid.kavrakilab.org/	Hall-Swan et al. (2021)
DeepR2cov	Discovery of potential agents for treating the excessive inflammatory response in COVID-19 patients by a deep representation on heterogeneous drug networks	https://github.com/pengsl-lab/DeepR2cov.git	Wang et al. (2021d)
CoV-AbDab	A coronavirus antibody database containing over 1400 published/patented antibodies and nanobodies	http://opig.stats.ox.ac.uk/webapps/coronavirus	Raybould et al. (2021)
SARS-CoV-2 3D	Supply and analysis of possible experimentally solved and created 3D structures of SARS-CoV-2	https://sars3d.com/	Alsulami et al. (2021)
CORDITE	Combination of state-of-the-art data on potential drugs against the SARS-CoV-2	https://cordite.mathematik.uni-marburg.de	Martin et al. (2020)
CoV3D	Resource for up-to-date coronavirus protein structures	https://cov3d.ibbr.umd.edu	Gowthaman et al. (2021)
DockCoV2	Prediction of the binding affinities of FDA-approved and taiwan national health insurance (NHI) drugs against specific targets	https://covirus.cc/drugs/	Chen et al. (2021)

### 3.2 Small-molecule inhibitors against the S protein

**FIGURE 9:**
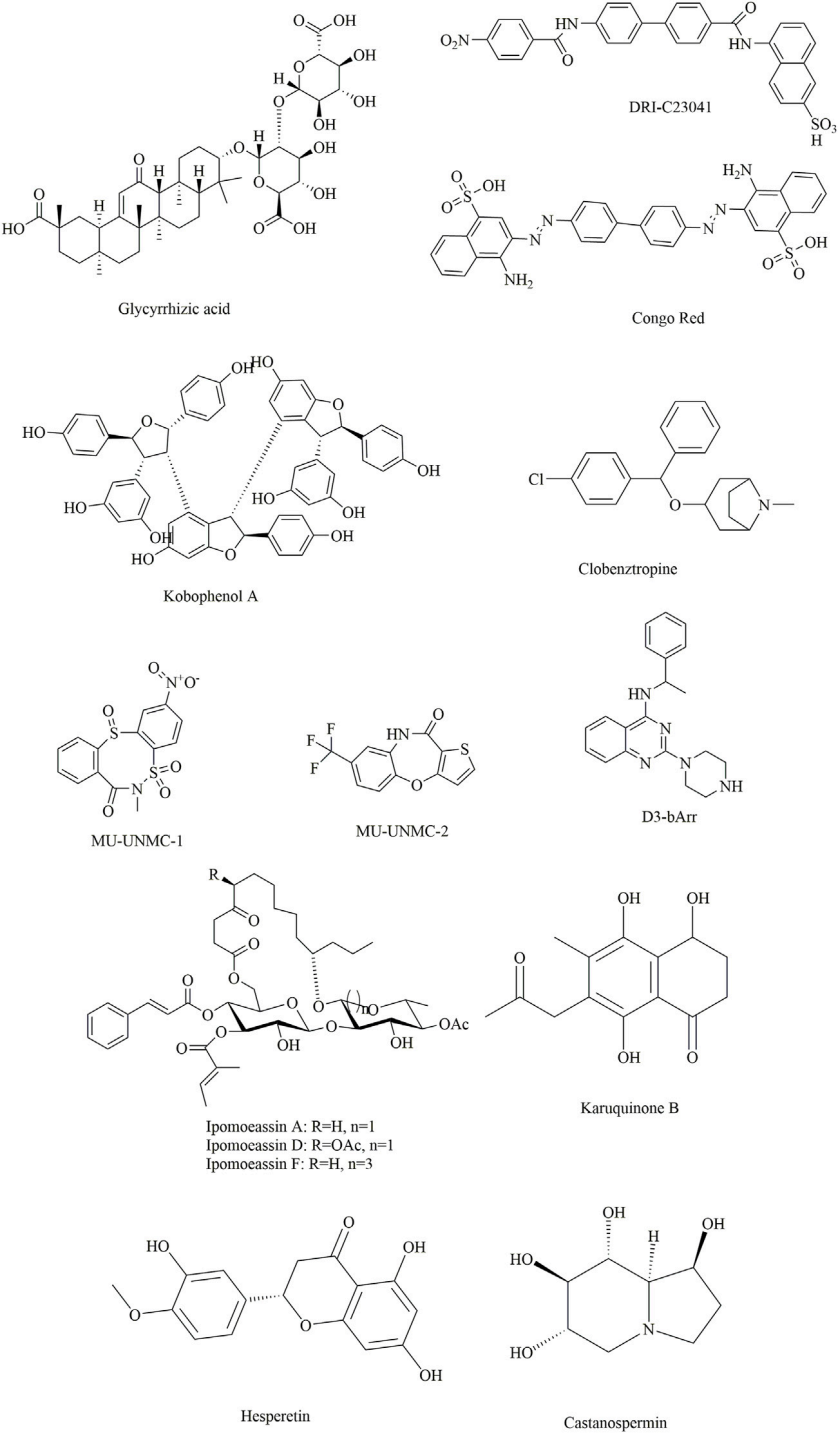
Several potential natural products showing inhibitory effects against SARS-CoV-2.

In attempts to discover and identify small-molecule inhibitors against the S protein, an efficient drug screening strategy is extremely important for structure-based, fragment-based, mechanism-based, and computer-guided drug discovery (Figure 9).

#### 3.2.1 Discovery of natural products for use as inhibitors against the S protein

Compared with neutralizing antibodies, small-molecule inhibitors might be more challenging for the blockade of RBD-hACE2 interactions due to the lack of well-defined binding pockets. However, they might offer alternatives that are more broadly active, more patient-friendly, less immunogenic, and more controllable than antibodies due to improved pharmacokinetics, stability, and dosage logistics (Song and Buchwald, 2015; Bojadzic and Buchwald, 2018; Xiu et al., 2020). Traditionally, natural products are important sources for novel drug development due to their rich sources, chemical diversity, large chemical space diversity, and biological activities. Therefore, natural products could make good starting points for modern drug design for the treatment of COVID-19 (Hu et al., 2021a; Mahmudpour et al., 2021). Together with the combination of computer-aided drug design and biological verification, drug development based on natural products is believed to be an efficient strategy for modern drug discovery.

Yu et al. applied this method to discover bioactive monomers from the active ingredient of licorice (*Glycyrrhiza uralensis* Fisch) for broad-spectrum anti-coronavirus candidates (Yu et al., 2021). In addition, surface plasmon resonance (SPR) assays, NanoBit assays and MTT assays were used simultaneously to determine the binding activities, inhibitory activities, and cell toxicities of selected compounds. The results showed that glycyrrhizic acid (ZZY-44) was an efficient (IC_50_: 22 μM) and broad-spectrum anti-coronavirus molecule with low toxicity (CC_50_ > 100 μM) *in vitro*, which could disrupt the interaction between the RBD and ACE2 (K_D_ = 0.87 μM).

Damir Bojadzic et al. (2021) identified several promising candidates by screening a compound library of organic dyes. Among them, Congo red, direct violet 1, Evans blue and novel drug-like compounds (DRI-C23041, DRI-C91005) showed inhibitory effects against the interaction of hACE2 with the spike protein with low micromolar activity (IC_50_: 0.2–3.0 μM). Notably, the results revealed that the inhibitors identified could bind the SARS-CoV-2-S protein but not hACE2, which provides great significance for the development of small-molecule inhibitors of PPIs critical for SARS-CoV-2 attachment/entry.

Suresh Gangadevi et al. discovered that kobophenol A is a potential inhibitor capable of blocking the interaction between the ACE2 receptor and S1-RBD by virtual screening of a library of natural compounds (Gangadevi et al., 2021). In this study, a computer-aided drug design strategy was applied to screen natural compounds, determine conformational changes and predict potential binding sites, including molecular docking and molecular Dynamic studies. The results showed that kobophenol A from *Caragana sinica* extract could disrupt the interaction between ACE2 and the SARS-CoV-2 S protein (IC_50_: 1.81 ± 0.04 μM and EC_50_: 71.6 μM). In addition, two potential binding sites for Kobophenol A were predicted, including the ACE2 hydrophobic pocket and the spike1/ACE2 interface.

Structure-based drug development is a proven method for high-throughput screening of specific compounds. In a study, through molecular Dynamic simulations and molecular docking, the hydrophobic pocket at the FP domain was first investigated, revealing the key binding regions (especially the FP hinge loop) and interactions. Then, a pharmacophore model was generated based on the predicted binding interaction. After that, nearly 200,000 drug-like compounds in the NCATS inhouse library were screened according to pharmacophore- and 3D-shape-based searches. Then, the 2,000 top-scoring compounds from docking were selected, clustered and visually inspected. Ultimately, 120 compounds were prioritized for further evaluation. This led to the discovery of two novel chemotypes of entry inhibitors (clobenztropine and D3-βArr), which displayed single-digit micromolar inhibition against SARS-CoV-2 (IC_50_: 12.6 and 15.8 μM) as well as SARS-CoV-1 and MERS (Hu et al., 2021b). It is interesting that although the two inhibitors are structurally distinct, they showed a similar binding mode at the fusion peptide (FP) domain, including an H-bond formed between Asp867 and the N atom of the polar headgroup and the π−π stacking interaction with Phe833. This further demonstrates the importance of the FP-binding site as a promising target for the structure-based development of novel inhibitors as drug candidates for treating COVID-19.

Similarly, two compounds (MU-UNMC-1 and MU-UNMC-2) were identified as being capable of blocking both SARS-CoV-2 replication at submicromolar IC_50_ values in human bronchial epithelial cells (0.67 and 1.72 µM) and Vero cells (5.35 and 1.63 µM) and the replication of rapidly transmitting variants of concern, including South African variant B.1.351 (IC_50_ = 9.27 and 3.00 mM) and Scotland variant B.1.222 (IC_50_ = 2.64 and 1.39 mM) (Acharya et al., 2021). In particular, MU-UNMC-2 could function synergistically with remdesivir (RDV), indicating that RDV and MU-UNMC-2 might be developed as a combination therapy to fight SARS-CoV-2.

Ipomoeassins A-E, as a new family of glycoresins, were isolated from the leaves of *Ipomoea squamosa* found in the Suriname rainforest in 2005 (Cao et al., 2005). They were shown to inhibit the proliferation of A2780 human ovarian cancer cells; among them, Ipomoeassin F (Ipom-F) is a potent natural cytotoxin that inhibits the growth of many tumour cell lines as a selective inhibitor of Sec61-mediated protein translocation at the ER membrane (Zong et al., 2019). However, in a recent study, it was found that Ipomoeassin-F could also inhibit the *in vitro* biogenesis of the SARS-CoV-2 spike protein (O'Keefe et al., 2021). It was also revealed that integration of the viral S protein and ACE2 into the endoplasmic reticulum membrane was significantly reduced by Ipom-F, while several other viral membrane proteins were unaffected.

In a study by Mathew (Al-Sehemi et al., 2020), 31,000 natural compounds of the natural product activity and species source (NPASS) library were screened for the discovery of special hits capable of interfering with the SARS-CoV-2 spike protein. The results showed that Castanospermine from a culture extract of *Fusarium solani* and Karuquinone B from different plant species (e.g., *Cassine glaucawere*) were identified and selected based on their binding affinity and pharmacokinetic data. However, no information is available regarding the antiviral activities of kuquinone B, and castanospermine was determined to show antiviral effects (Chang et al., 2013) against various viruses *in vitro* and *in vivo,* such as Ebola (Dowall et al., 2016) and Zika (Bhushan et al., 2020). Similar to Anamika et al. (Basu et al., 2020), the natural products hesperidin, emodin and chrysin were found to be capable of inhibiting SARS-CoV-2. In particular, hesperidin from *Citrus aurantium* could interfere with the interactions between ACE2 and the spike protein. In addition, its interaction was predicted to be located in the middle shallow part of the surface of RBD of Spike, in which the dihydroflavone part was parallel with the β-6 sheet of RBD and the sugar part was inserted into the binding site in the direction away from ACE2 (Wu et al., 2020).

#### 3.2.2 Drug repurposing for inhibitors against the S protein

**FIGURE 10:**
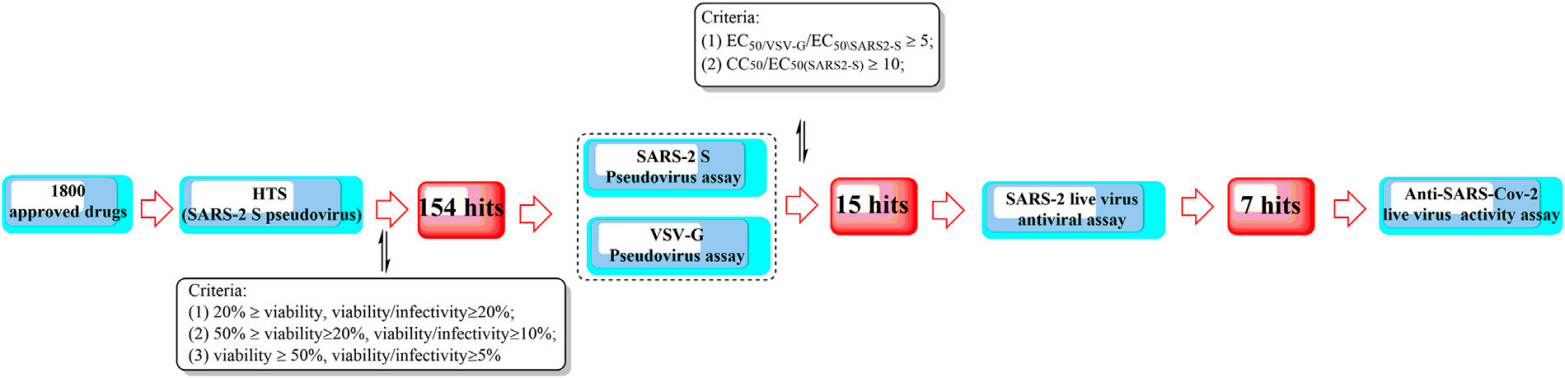
A high-throughput screening method for the efficient discovery of SARS-CoV-2 entry inhibitors using a SARS2-S pseudotyped virus.

Recently, Yang et al. proposed a high-throughput screening method for the efficient discovery of SARS-CoV-2 virus entry inhibitors using SARS2-S pseudotyped virus (Figure 10) (Yang et al., 2021b). The results showed that 7 drugs could significantly inhibit SARS2 replication and reduce supernatant viral RNA load with a promising level of activity. Among them, trimeprazine, azelastine hydrochloride, and clemastine, classified as histamine receptor antagonists with clemastine, were determined to show the strongest anti-SARS2 activity. In addition, clemastine is capable of targeting the sigma 1 and sigma 2 receptors (Gordon et al., 2020). Sigma-1 and sigma-2 are endoplasmic binding sites, and sigma-1 is usually considered as a pluripotent chaperone for regulating Ca^++^ fluxes (Ortega-Roldan et al., 2013) and the K^+^ channels (Abraham et al., 2019). In addition, studies also showed that neuroprotection, neuroregulation, and modulation of the proliferative status of cells might also be associated with the functions of sigma 1 (Abate et al., 2020). On the contrary, sigma-2 might play roles in regulating cell death (Pati et al., 2017). Therefore, attention should be given to antihistamine drugs for the development of antiviral agents.

**FIGURE 11:**
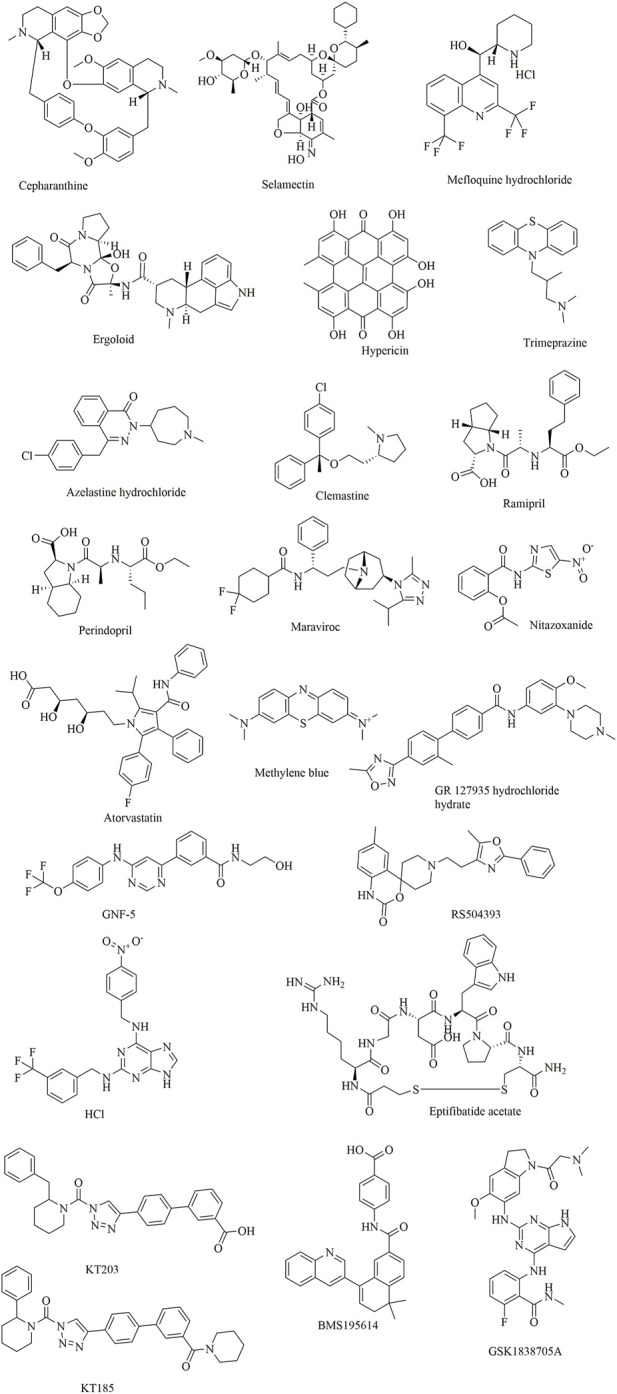
Clinically approved drugs repurposed for the treatment of COVID-19.

In a study by Smith et al. (2020), SUMMIT, the world’s most powerful supercomputer, was applied for the identification of approved drugs that could bind to either the S-protein receptor recognition region or the S protein-human ACE2 interface. Additionally, in this study, an ensemble virtual high-throughput screening docking strategy in combination with restrained temperature replica-exchange molecular dynamic (restrained T-REMD) simulations was used. The results showed that 77 hits (24 having official approval) from over 8,000 drugs, metabolites, and natural products were found to be capable of binding efficiently to the target. Among them, the three top-scoring ligands (cepharanthine, ergoloid, and hypericin) have ZINC15 annotations; however, no experimental testing has been reported regarding the effectiveness of the identified drugs.

In addition to computer-aided drug discovery, other novel and efficient tools for the rapid screening of SARS-CoV-2 inhibitors must also be established. To achieve this, an electrochemical impedance spectroscopy (EIS)-based biosensor was designed and characterized (Kiew et al., 2021). In this system, the core sensing element mainly consisted of a recombinant ACE2 protein-coated palladium nanothin-film (ACE2-Pd-NTF) electrode, which could be used to detect alterations occurring in the binding of S-protein to ACE2 when exposed to the test molecules. With this method, several potential pharmacological leads that could disturb SARS-CoV-2-ACE2 binding were successfully identified, such as ramipril and perindopril. Although it is yet to be fully explored at present, this approach has good potential for becoming a mainstream approach for efficient, timesaving, and cost-effective drug discovery and repurposing in the future.

In another study, it was found that maraviroc, FTY720, nitazoxanide and atorvastatin could inhibit SARS-CoV-2 replication in cell culture by screening 19 small molecules and 3 biologics (Risner et al., 2020). However, confocal microscopy with overexpressed S protein revealed that maraviroc reduced the extent of S protein-mediated cell fusion.

Considering the huge chemical space of organic dyes, it is believed that small-molecule inhibitors for PPIs would be more likely to be discovered in such compounds (Downing et al., 2017; Bojadzic and Buchwald, 2018). In a recent study, methylene blue (MB) was found to be capable of inhibiting the interaction between the S protein and ACE2 in a concentration-dependent manner (IC_50_ = 3–3.5 μM), even in the absence of light (Bojadzic et al., 2020). In another study, nonphotoactivated MB showed *in vitro* activity at a very low micromolar range with an EC_50_ of 0.30 ± 0.03 μM and an EC_90_ of 0.75 ± 0.21 μM at a multiplicity of infection of 0.25 against SARS-CoV-2 (strain IHUMI-3) (Gendrot et al., 2020). As a tricyclic phenothiazine compound, it was approved by the FDA for the treatment of methemoglobinemia. However, MeBlu shows dose-dependent toxicity, with symptoms including nausea, vomiting, and haemolysis, when used at doses >2 mg/kg (Dabholkar et al., 2021). In addition to disturbing the direct interaction between SARS-CoV-2 spike protein and ACE2, MB was reported to play various biological roles in blocking the entry of SARS-CoV-2 into the cells, such as preventing the endocytosis of virions into the cells by increasing endosomal and lysosomal intracellular pH and inhibiting the intermediate stages of endocytosis; blocking the formation of the NLRP3 complex to prevent the cytokine storm (van den Berg and Te Velde, 2020); inhibiting nitric oxide synthase and promoting saturation of oxygen to terminate the effects of bradykinin (Ghahestani et al., 2020; Karamyan, 2021).

Shweta et al. investigated FDA-approved LOPAC library drugs against both the RBD of the spike protein and the ACE2 host cell receptor with a high-throughput virtual screening approach and molecular simulations (Choudhary et al., 2020). The results showed that GR 127935 hydrochloride hydrate, GNF-5, RS504393, TNP, and eptifibatide acetate were capable of binding to the ACE2 receptor. In addition, KT203, BMS195614, KT185, RS504393, and GSK1838705A could bind to the RBD of the spike protein.

In a recent study, repurposing clinically approved drugs for the treatment of COVID-19 in a 2019-nCoV-related coronavirus model was achieved (Fan et al., 2020). The results showed that cepharanthine (CEP), selamectin, and mefloquine hydrochloride exhibited complete inhibition of cytopathic effects in cell culture at 10 mmol/L. In particular, CEP displayed the most potent inhibition of GX_P2V infection (EC_50_ = 0.98 mmol/L). In another study using Calu-3 cells, CEP was also determined to show an inhibitory effect against SARS-CoV-2, with an IC_50_ of 30 μM (as opposed to an IC_50_ of 4.47 μM in Vero cells) (Jeon et al., 2020; Ko et al., 2020). CEP, a Japanese-approved alopecia drug, is an alkaloid used frequently to treat radiation-induced leukopenia, exudative middle ear catarrh, and viper bite. Transcriptome analysis indicated that CEP could efficiently reverse most dysregulated genes and pathways in infected cells, such as the ER stress/unfolded protein response and HSF1-mediated heat shock response (Li et al., 2021).

#### 3.2.3 Novel drug development for inhibitors against the S protein

**FIGURE 12:**
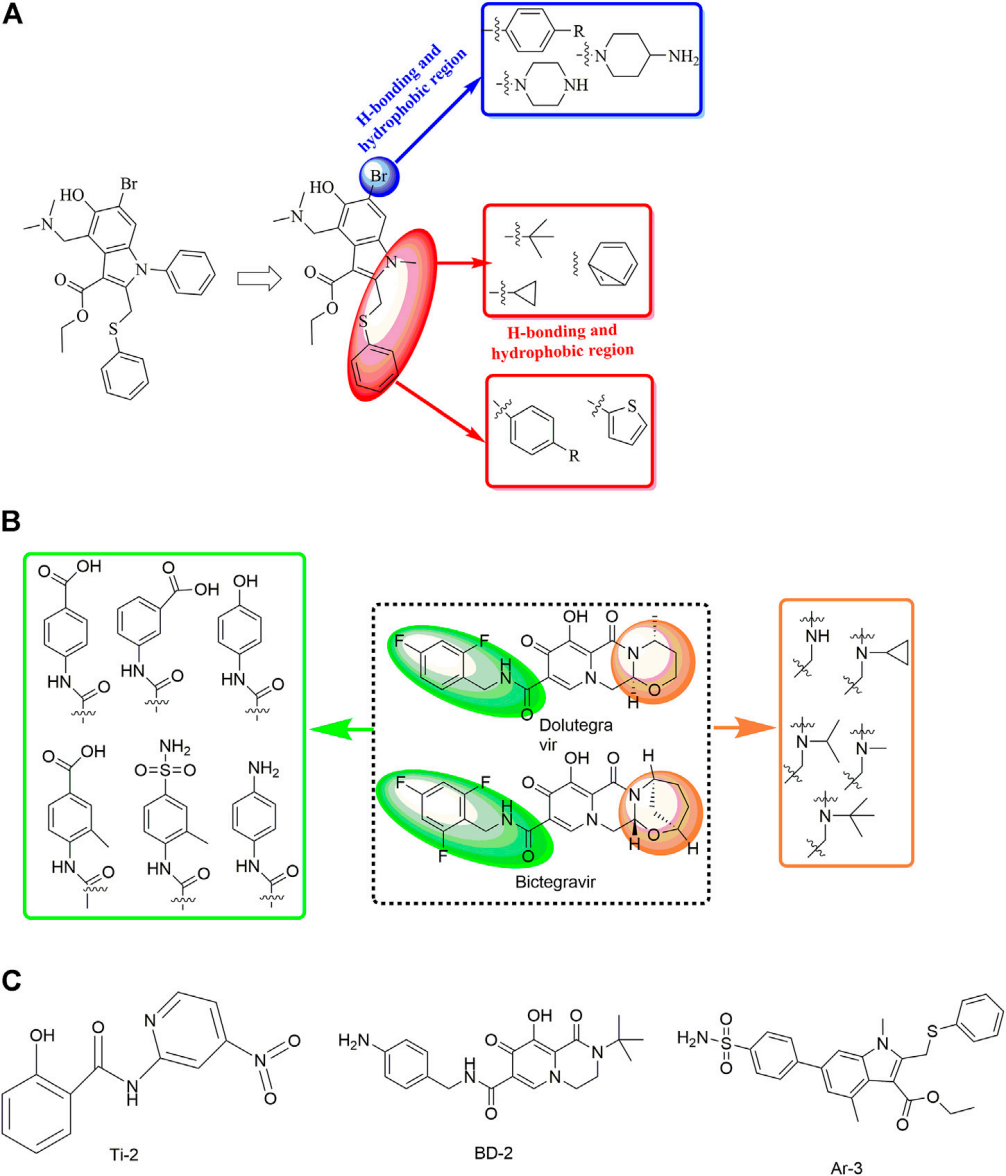
Strategies for the further structural optimization of arbidol **(A)**, dolutegravir and bictegravir **(B)** and the structures of BD-2, Ti-2 and Ar-3.

In a study by Sun et al. (2021), the discovery and rational design of small-molecule inhibitors of the SARS-CoV-2 S protein was achieved (Figure 12). First, molecular docking with the Lamarckian genetic algorithm was applied for the screening of 14 antiviral molecules by analysing the binding energy and interactions between the ligands and the receptor, the SARS-CoV-2 S protein. This approach led to the discovery of tizoxanide, dolutegravir, bictegravir, and arbidol, which have high binding energies and are capable of binding to the S1 and S2 subunits. Then, structure-based rational design was performed using the molecular connection method and a bioisosterism strategy by introducing specific functional groups to enhance the binding energies and interactions with the S protein. In this way, Ti-2, BD-2, and Ar-3 were identified with much stronger binding ability to the S protein. Although no experimental data about the antiviral activities have been reported to date, the strategy used in this study might be valuable in the rational design of novel anti-SARS-CoV-2 drugs.

**FIGURE 13:**
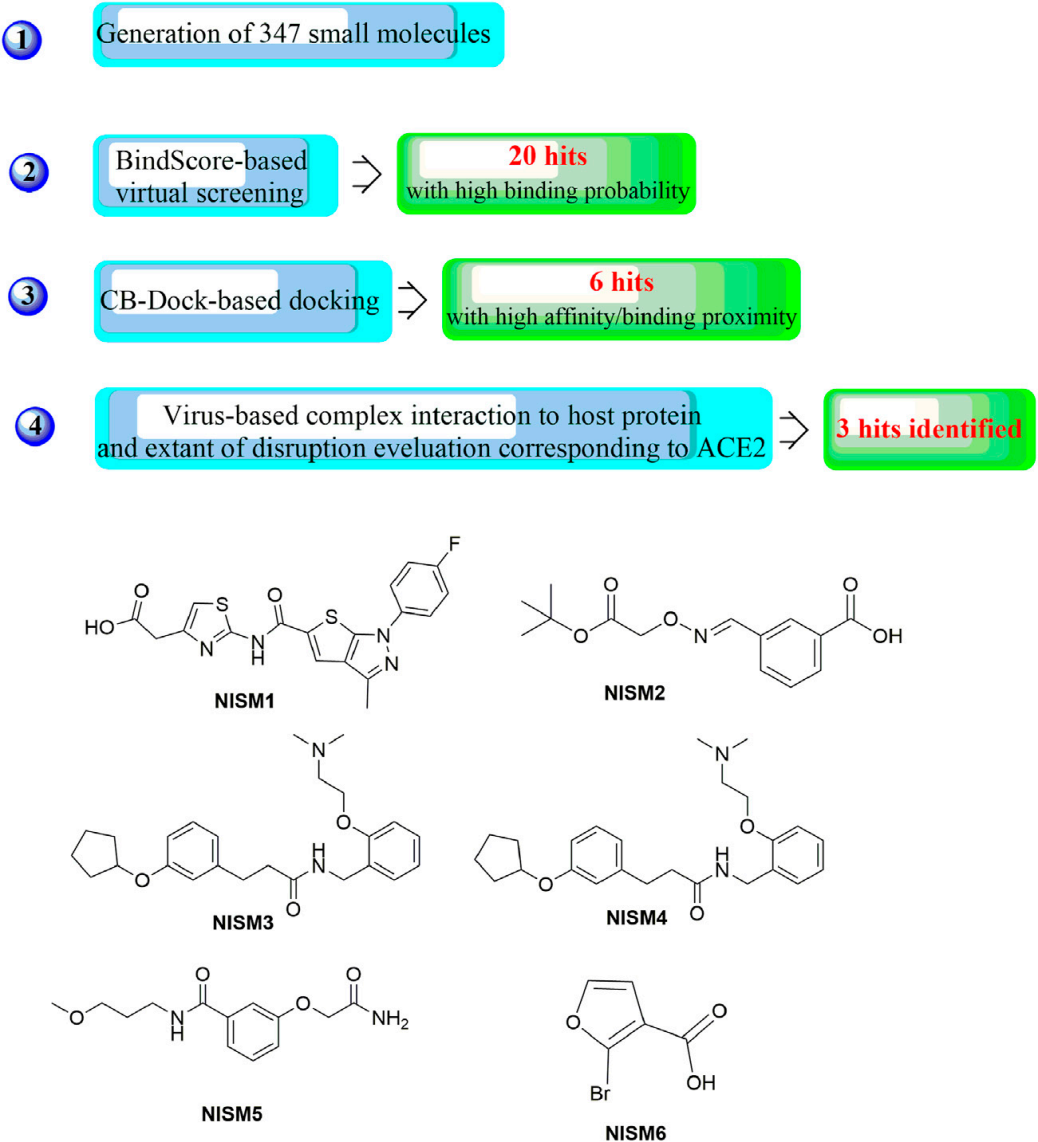
Strategies for the further structural optimization of arbidol **(A)**, dolutegravir and bictegravir **(B)** and the structures of BD-2, Ti-2 and Ar-3.

In a recent study, another strategy was proposed for the rational design of novel inhibitors against the binding of SARS-CoV-2 to the ACE2 receptor (Figure 13) (Mishra and Nandi, 2021). First, favourable and accessible binding pockets of the RBD were established using the deep convolution neural network (DCNN) model based on parameters such as the hydrogen bond acceptor/donor, hydrophobicity, and ionization energy. Based on the four different binding pockets obtained, decoded *de novo* drug molecules were then generated with a shape-captioning network, leading to the formation of a total of 347 ligands in the simplified molecular input line entry system (SMILES) strings. BindScope based on a DCNN was then used for virtual high-throughput screening according to the simulated binding probabilities, which resulted in the identification of the top 20 molecules with high probabilities of binding. The obtained 20 hits were then subjected to further detailed screening using the CB-Dock server with AutoDock Vina, and 6 compounds showing the best potential to inhibit the spike protein–ACE2 interaction were ultimately identified. Although the newly designed compounds lack antiviral activities, they possess excellent drug scores and are nontoxic and nonmutagenic compared with the several existing antiviral drugs available on the market. Similarly, Rituparno et al. developed an atomistic *de novo* inhibitor generation-guided drug repurposing strategy based on the free-energy validation by well-tempered metadynamics (Chowdhury et al., 2021). It contains three main steps including: generation of new molecules, structural similarity mapping and validation of the binding abilities, well-tempered metadynamics free energy calculations.

**FIGURE 14:**
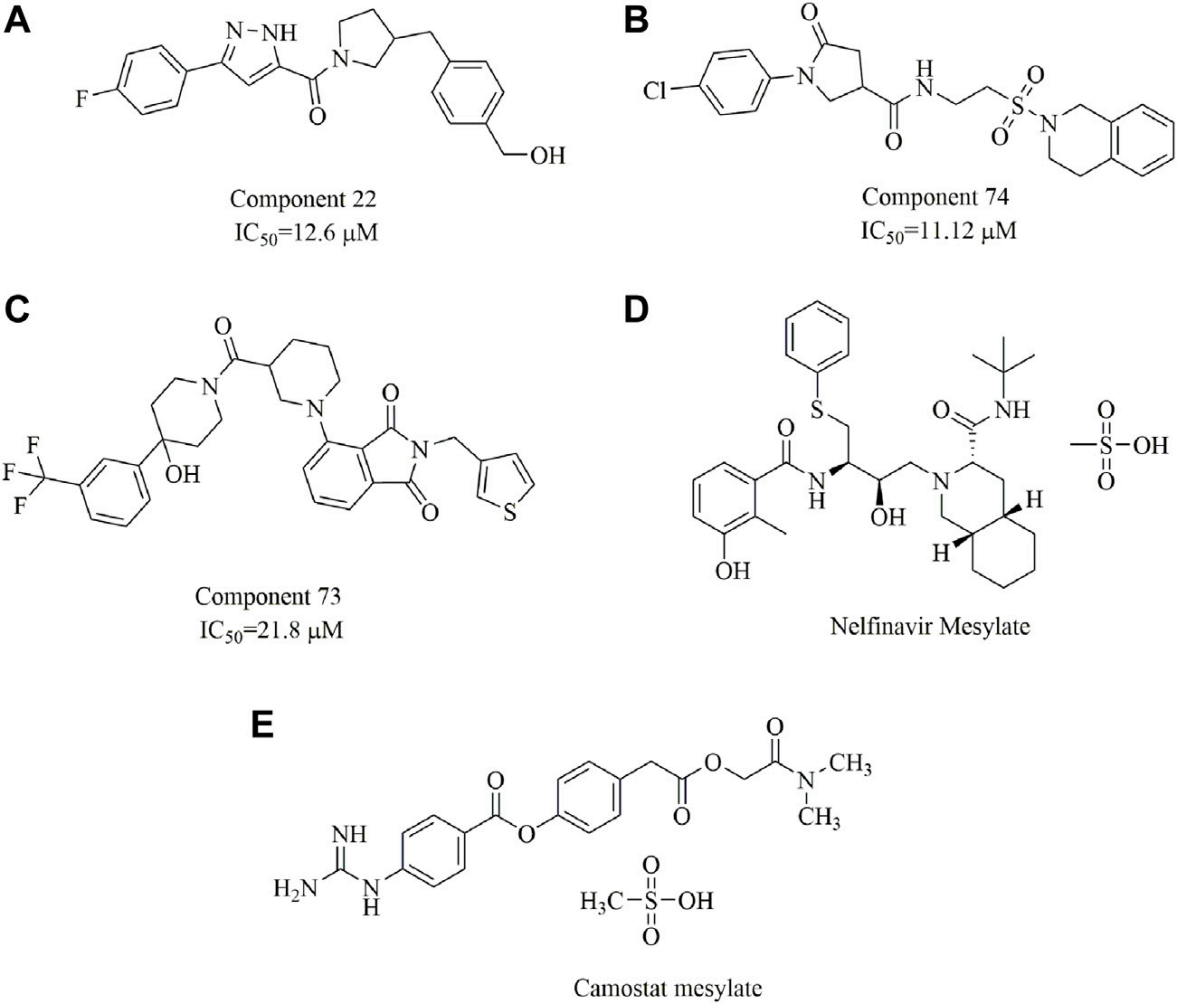
Chemical structures of the identified small-molecule fusion inhibitors against MERS-CoV and their inhibitory properties.

In one study, three small-molecule fusion inhibitors with potent inhibitory activity against MERS-CoV were identified (Figures 14A–C); these inhibitors could bind to the surface of HR1, interfering with HR2 recognition of HR1 (Kandeel et al., 2020). These compounds are considered the first generation of MERS-CoV small-molecule fusion inhibitors. In another study (Musarrat et al., 2020), nelfinavir mesylate (Viracept, an anti‐HIV drug, Figure 14D) was found to be a potent inhibitor of cell fusion caused by the SARS-CoV‐2 S protein, with complete inhibition even at 10 μM. Markus et al. discovered that the S protein of SARS-CoV-2 is cleaved by the serine protease TMPRSS2 and that cell entry could be inhibited by the clinically proven protease inhibitor camostat mesylate (Figure 14E (Hoffmann et al., 2020). Additionally, ligands have been suggested to bind to the interfaces of the trimeric structure of the SARS-CoV-2 S protein and may destabilize the quaternary S protein structure, thereby interfering with the SARS-CoV-2 life cycle (Bongini et al., 2020). In our opinion, this is of importance for the discovery of promising drug candidates but requires evidence-based support.

#### 3.2.4 Application of machine learning for COVID-19 drug discovery

Machine learning (ML) and deep learning (DL) algorithms as two of the most widely used artificial intelligence technology could also be applied to predict drug-target interactions and then validate the predicted drugs in terms of chemical, biological, and physical characteristics based on the various predictive models (Patel et al., 2020; Crampon et al., 2022). To date, two main types of ML algorithms have been developed including: supervised learning (from training samples with known labels) and unsupervised learning (from training samples without known labels) (Rifaioglu et al., 2019). In a study by Batra et al. (2020), a rational screening strategy was developed combining machine learning-based models and high-fidelity ensemble docking studies. Firstly, viable targets for drug discovery wase determined as SARS-CoV-2 S-protein at its host receptor region or the S-protein: human ACE2 interface. Then, the Vina scores were estimated by random forest (RF) regression models for the construction of molecular descriptors, which were applied to represent the molecules for the development of the ML models. The validated ML models were then used for virtual screening ligands from drug and biomolecule data sets. Top scoring 187 hits (75 FDA-approved) were further validated by all atom docking studies, and important molecular descriptors and promising chemical fragments are identified to guide future experiments. Coveney et al. designed a novel *in silico* method for drug design by coupling ML with physics-based (PB) simulations (Bhati et al., 2021). The accurate PB simulations would make the drug design process smarter by calculating the binding free energies of obtained hits from the output of a deep learning (DL) algorithm, which will then fed back to the DL algorithm to improve its predictive performance. Recently, a machine-learning method was proposed capable of identifying drug mechanism of actions based on the cell image features (Han et al., 2021). In this method, the supervised information theoretic metric-learning (ITML) algorithm was used for converting the characteristics of drugs with similar mechanism of actions clustered by affinity propagation algorithm. Therefore, this method would be more useful in the development of candidates with similar action mechanisms.

Undoubtedly, these results clearly demonstrate the power and efficiency of the ML-based screening. However, in such studies, development of accurate and reliable ML models is the key part of a successful ML-based strategy (Lv et al., 2021), including the data quality and algorithm design. Therefore, it is important to train and validate the models over established data sets.

## 4 Conclusion and perspectives

Since 2019, the outbreak of SARS-CoV-2 has posed a global health emergency. The high morbidity and mortality associated with COVID-19, especially the lack of an approved efficient drug or vaccine for SARS-CoV-2, presents the urgent need for developing standard antiviral therapies. Drug development to counter COVID-19 could be streamlined by targeting different viral proteins, especially the S protein, which is an effective choice to interfere with viral entry into host cells. Computer-aided modern drug development provides a time- and effort-saving alternative for hit identification, lead optimization and rational drug design. However, just as every coin has two sides, computer-aided drug development also has its own disadvantages. Virtual screening aided by structure-based docking has inherent deficiencies caused by various factors, such as the lack of crystal structures of target proteins and the influence of various conformations and pockets, which could lead to false-negative results. Hence, improving the performance and the accuracy of the computational resources to streamline the workflow used is still needed. In addition, to maximum full play the function of the computational resources, deeper insights into the Spike protein structure, function, and interactions with ACE2 is still essential. Especially, it has demonstrated that specific mutations in the S protein will greatly influence its infectivity, transmissibility, virulence. Therefore, more intensively studies on the adaptive evolutionary mechanisms will further help develop proper and more efficient strategies to fight SARS-CoV-2.

To date, most studies focused on novel drug development for the prevention and treatment of COVID-19 are usually at the *in vitro* experimental stage in the absence of actual *in vivo* data. This might also result in the discovery of nonfunctional ligands in animal or *in vivo* experiments. Therefore, a more efficient strategy should also be investigated by integrating computational resources with *in vivo* experiments. In this way, it would to the greatest extent avoid the false positives and thereby maximize the odds of success in following development process.

Recently, multitarget drugs (MTDs) have attracted great attention due to their advantages in the treatment of complex diseases such as Alzheimer’s disease. Since ACE2 is a multifunctional protein, MTDs targeting several sub-pathologies simultaneously might present a better approach for the treatment of COVID-19. While it is conceivable that rational design of MTDs with excellent performance against SARS-CoV-2 is a huge challenge for medicinal chemists; it demonstrates great potential and provides a promising method for treating complex diseases, including COVID-19.

Significantly, it has been reported that phospholipidosis was a common mechanism underlying the antiviral activity of many repurposed drugs (Tummino et al., 2021). Therefore, one the one hand as mentioned above, adequate, and timely *in vitro* tests would be more important for the detection of phospholipidosis and elimination of the identified false positives in early drug discovery. On the other hand, to avoid phospholipidosis, drug discovery or screening of antiviral drugs should be focused more on the target-directed mechanism as highlighted in this review.

In summary, this review is expected to provide a potential framework for designing and developing promising anti-SARS-CoV-2 therapeutics.
